# Genetic variation in the glymphatic pathway predicts cognition and neurodegeneration in preclinical Alzheimer’s disease

**DOI:** 10.21203/rs.3.rs-9444687/v1

**Published:** 2026-05-06

**Authors:** Ayeisha Milligan Armstrong, Eleanor K. O’Brien, Shane M. Fernandez, Vincent Doré, Pierrick Bourgeat, Rosita Shishegar, Paul Maruff, Christopher C. Rowe, Victor L. Villemagne, the Alzheimer’s Disease Neuroimaging Initiative, the AIBL Research Group, Tenielle Porter, Simon M. Laws

**Affiliations:** Edith Cowan University; Edith Cowan University; Edith Cowan University; Commonwealth Scientific and Industrial Research Organisation; Commonwealth Scientific and Industrial Research Organisation; Commonwealth Scientific and Industrial Research Organisation; Cogstate (Australia); Austin Health; University of Pittsburgh; University of California, San Francisco; Florey Institute of Neuroscience and Mental Health; Edith Cowan University; Edith Cowan University

**Keywords:** Genetic variation, genetic risk scores, glymphatic pathway, aquaporin-4, dystrophin-associated complex, Amyloid-β, cognition, brain volume, neurodegeneration

## Abstract

**Background:**

Impaired glymphatic clearance has been implicated in Alzheimer’s disease (AD) through reduced clearance of amyloid-β (Aβ) and other metabolites from the brain. Mislocalisation of aquaporin-4 (AQP4), a water channel protein anchored to astrocytic endfeet by the dystrophin-associated complex (DAC), has been linked to increased Aβ accumulation, neurodegeneration and cognitive impairment. In animal models, genetic ablation of DAC subunits, leading to AQP4 mislocalisation, increases Aβ accumulation. Genetic variation in *AQP4* has been examined in the context of AD, but variation in key DAC genes has not been systematically investigated in humans. This study examined whether variation within glymphatic pathway genes is associated with AD-related phenotypes in individuals on the AD trajectory.

**Methods:**

Associations between genetic variation in glymphatic pathway-related genes (*AQP4* and three DAC genes: *DAG1*, *DTNA*, and *SNTA1*) and brain Aβ burden, cognition and regional brain volumes were analysed in two longitudinal cohorts of cognitively unimpaired older adults who were Aβ-positive or demonstrated ongoing Aβ accumulation. Linear regression models assessed cross-sectional and longitudinal outcomes within each cohort, followed by a meta-analysis. Pathway-based glymphatic genetic risk scores (G-GRS) were constructed to assess cumulative genetic impact.

**Results:**

At the single-variant level, *AQP4*, *DTNA*, and *DAG1* showed significant associations with cognition, grey matter atrophy, and ventricular volume after correction for multiple testing. Variants in *AQP4* (rs10502478 and rs10164026) were associated with differences in cross-sectional PACC scores. Whilst rs45556134 within *DTNA* was associated with grey matter atrophy. Further, six variants (rs13079082 *DAG1*, rs8092794, rs17642885, rs7233779, rs78837792 and rs79500711 *DTNA*) were associated with cross-sectional ventricular volumes. At the polygenic level, higher G-GRS were associated with lower grey matter volume, faster grey matter atrophy, larger ventricular volumes and lower cognitive composite scores.

**Conclusions:**

Variation within key glymphatic pathway genes is associated with early differences in cognition and neurodegeneration-related brain measures in cognitively unimpaired Aβ-positive older adults. These findings support a contributory role for glymphatic pathway dysfunction in early AD-related brain vulnerability and identify glymphatic pathway genetic risk as a potential marker for risk stratification. A limitation of this study is the absence of direct experimental measures of glymphatic clearance function, which will be required to confirm the functional impact of these genetic findings.

## Background

Astrocytes are brain glial cells that mediate the regulation of synapses, provide metabolites and modulate signalling to neurons, maintain the blood-brain barrier (BBB), and regulate neuroinflammation (1). Astrocytic morphology and function are altered in the neurodegenerative processes characteristic of Alzheimer’s disease (AD) (2, 3). For example, while reactive astrogliosis has been shown to provide a protective phenotype in response to brain insult, this response is paradoxically detrimental in AD mouse models (4–6). Astrogliosis is considered an early event in the AD cascade, with human and animal studies indicating that astrocytic changes occur during the prodromal phase and before amyloid-β (Aβ) plaque formation (6, 30, 31). Single-cell transcriptomics data from human post-mortem brain tissue have shown that changes in astrocytic gene expression profiles across the AD trajectory are associated with pTau to Tau ratios, Aβ load and also differ by brain region (7), which may account the paradoxical effects seen in AD (6). Some functional impairments of astrocytes in AD include dysregulated calcium signalling and impaired glutamate buffering (8–10). In addition, astrocytes are involved in the degradation and removal of Aβ from the brain via the astrocytic endfeet, which envelope the brain’s vascular system (11). Astrocytic endfeet are a central component of the the postulated glymphatic system, which describes a brain system involved in the removal of waste products such as Aβ. The system involves the mixing of cerebrospinal fluid (CSF) and interstitial fluid through the perivascular network, with subsequent drainage from the brain to the cervical lymphatic system (12).

In humans, the glymphatic pathway is dependent upon the aquaporin-4 (AQP4) water channel protein, which is expressed at the astrocytic endfeet (13, 14). Loss of perivascular AQP4 localisation is observed in AD and is strongly associated with increasing accumulation of Aβ and tau (15). Consistent with these observations in humans with AD, *AQP4* knock-out mice models show greater Aβ accumulation, increased astrogliosis, disturbances in proteins important for synapses and increased cognitive impairment (16). Another vital component of the astrocytic endfeet is the dystrophin-associated complex (DAC), which is a scaffolding multi-protein complex that anchors AQP4 to the astrocytic endfeet, and comprises α-syntrophin (SNTA1), dystroglycan (DAG1), dystrobrevin (DTNA) and dystrophin (DMD) proteins (17–20). The importance of the DAC in anchoring AQP4 for proper localisation at the astrocytic endfeet has been confirmed in animal models, where knock-out of components such as *DMD* and *SNTA1* results in disruptions to AQP4 perivascular localisation and reduced endfoot integrity (17, 19, 21, 22). Animal studies modelling the effects of mislocalisation of AQP4 have used *SNTA1* knock-out models to disrupt the DAC assembly and have shown increased Aβ accumulation in the brain, likely due to the inability to be cleared from the brain through the glymphatic system (23, 24). Post-mortem neuropathological studies in humans with AD show that mislocalisation of AQP4 away from astrocytic endfeet is associated with increasing Aβ burden, neurofibrillary tangles, and cognitive decline prior to the onset of dementia (24). Furthermore, studies in humans with clinically defined dementia show altered expression of the DAC components *DMD, DAG1*, and that altered expression of *DTNA* and *SNTA1* is associated with increased levels of phosphorylated tau within the temporal cortex (25). Together, these observations highlight the importance of examining genetic variants within the DAC sub-unit proteins both independently and in combination as a pathway score, as it is likely that the components are interdependent in ensuring proper AQP4 localisation and glymphatic pathway function.

Previous studies have identified relationships between genetic variants within *AQP4* and progression of cognitive decline in multiple neurodegenerative states and with brain Aβ burden (26–29). Therefore, understanding how variation in other genes within the glymphatic pathway contributes to neurodegeneration and cognitive decline represents an important next step. However, variants within genes encoding DAC subunits have not been studied extensively in humans. The primary aim of this study was to investigate whether variants in genes involved in the astrocytic-mediated glymphatic clearance system (*AQP4* and DAC genes) are associated with AD-related phenotypes (brain Αβ burden, cognition and regional brain volumes) in two independent cohorts of cognitively unimpaired older individuals. Cognitively unimpaired individuals were selected because astrogliosis is an early feature of AD pathogenesis (6, 30, 31), enabling the identification of early biological signatures of glymphatic dysfunction prior to overt neurodegeneration or clinical symptom onset. Given the strong link between the glymphatic system and Aβ clearance, cohorts were stratified by Aβ status to focus analyses on individuals most likely to exhibit glymphatic pathway-related vulnerability and to reduce confounding by Aβ pathology, and analyses focused on individuals who were Aβ-positive or on an Aβ-accumulating trajectory (Aβ^+^). A second aim was to assess the cumulative effects of genetic variants across glymphatic pathway genes through the construction and testing of biological pathway-informed genetic risk scores directed at these AD-related phenotypes.

## Methods

### Study Participants

Participant data from two extensively characterised longitudinal cohort studies, the Australian Imaging Biomarker and Lifestyle (AIBL) Study of Ageing (https://aibl.org.au/) and the North American Alzheimer’s Disease Neuroimaging Initiative (ADNI), (www.adni-info.org) were used (32–36). Both AIBL and ADNI were designed to assess biomarkers and cognitive measures linked to disease progression. Initiated in 2006, the AIBL study was developed to align closely with ADNI, which began in 2003, by using similar methods to support meaningful comparisons between the two cohorts. Participant inclusion in the current study was limited to those assessed as cognitively unimpaired (CU) at baseline, based on comprehensive neuropsychological evaluations across multiple cognitive domains within each study cohort (32, 33, 36). The cohorts were then further stratified by brain Aβ levels estimated by positron emission tomography (PET). Participants were classified as Aβ positive (Aβ^+^) if they had a baseline Centiloid (CL) score (described below) > 20 or a positive slope of change over a minimum of three timepoints (indicative of Aβ accumulation). The remaining participants were classified as Aβ negative having CL ≤ 20 and no evidence of accumulation. The Aβ^+^ participants were the main focus of this study. In addition, participants were required to have genetic and appropriate covariate data available. Due to different outcome measures being assessed, the number of participants included in each analysis differed depending on data availability. Both AIBL and ADNI studies have ethics approval from their respective institutional ethics committees.

### Cognitive Assessment

The Preclinical Alzheimer’s Cognitive Composite (PACC) was used as the measure of cognition in the current study (37). The AIBL PACC and ADNI PACC were structured to evaluate the same cognitive domains (episodic memory, timed executive function, and mental status) using the cognitive assessments administered in each study. These assessments measure the same aspects of cognition, and they have been shown to be neuropsychologically equivalent in terms of their sensitivity to clinical disease progression in each cohort (37) .

### Brain Imaging Methods

Brain Aβ-burden was estimated using positron emission tomography (PET) with various tracers targeting Aβ. CapAIBL software was used to analyse the brain Aβ PET scans (38) and generate standardised uptake value ratios (SUVR) for all tracers. Centiloid values were subsequently derived from these SUVR (39, 40).

Magnetic resonance imaging (MRI) scans were used to estimate region-specific brain volumes at T1 using the magnetisation-prepared rapid gradient echo (MPRAGE) protocol. CurAIBL was used to estimate grey matter, white matter, hippocampal and ventricular volumes as previously described (41–43). Hippocampal volume refers to the average of participants’ left and right hippocampal volumes. Region-specific brain volumes were corrected for both intracranial volume and MRI scanner used prior to analyses.

### Genotyping and SNP selection

Genomic DNA from AIBL and ADNI participants was extracted from whole blood using QIAamp DNA blood spin column kits (Qiagen, Valencia, CA, USA), according to established protocols (32, 33, 35). In the ADNI cohort, Apolipoprotein E *(APOE)* genotypes were determined through PCR amplification, followed by digestion with the *HhaI* restriction enzyme and separation on a 4% Metaphor Gel for visualisation (44, 45). *APOE* genotyping for AIBL participants was conducted using TaqMan^®^ assays (Life Technologies, USA) on the QuantStudio^™^ 12K Flex Real-Time PCR System (Applied Biosystems^™^, USA), as previously described (46–48). *APOE ε4* status was included as a covariate as a binary variable, indicating the presence or absence of the *ε4* allele.

In the AIBL cohort, genome-wide single-nucleotide polymorphism (SNP) genotype data were generated using the Axiom Precision Medicine Diversity Array (Applied Biosystems^™^). For ADNI participants, genotyping was performed with various platforms, including the Illumina Human610-Quad BeadChip, Illumina HumanOmniExpress BeadChip, Illumina Infinium Global Screening Array v2 (GSA2), and Illumina Omni 2.5M BeadChip (Illumina, Inc., USA). The raw genotype data for both cohorts underwent imputation using the Trans-Omics for Precision Medicine (TOPMed) panel via the TOPMed Imputation Server (49, 50).

The DAC-associated genes *(SNTA1, DTNA* and *DAG1*) were included in the current analysis, while *DMD* was excluded due to being located on the X chromosome. SNPs were extracted from the four genes and their flanking regions (+/−10 kbp) and restricted to those available in both the AIBL and ADNI genetic datasets. Following this, SNP selection was undertaken after quality control (QC) measures and a fine-mapping approach was conducted on the ADNI genetic dataset. SNPs were excluded during QC if they had a call rate < 95%, minor allele frequency < 0.05 or deviated from Hardy Weinberg Equilibrium. Linkage disequilibrium pruning (window size = 50 kb, step size = 5, r^2^ = 0.8) was then performed, resulting in 137 SNPs that were included in the analysis.

### Statistical Analysis

All statistical analyses were undertaken using R Statistical Software Version 3.18 (51) (run in RStudio (1.2.1335) (52)), PLINK (v1.90b6.24) (53), METAL (54) or PRSice (55). Demographic variable summary statistics, including mean (standard deviation) and n (percentage), were examined for AIBL and ADNI participants.

To assess the relationship between SNPs and the outcomes measured (brain Aβ, regional brain volumes and PACC), linear regression analyses were undertaken separately for the two cohorts using PLINK (53). SNPs were assessed under additive, dominant and recessive genetic models. When assessing outcomes cross-sectionally, participants baseline data was examined. Covariates for the cross-sectional analyses included age, sex and *APOE-ε4* status. Additionally, when cognition was assessed as the outcome variable, years of education was also included as a covariate. For longitudinal assessment of regional brain volumes or PACC, a two-step approach was employed. Individual participants’ slopes and intercepts were estimated for each regional brain volume and PACC outcomes using linear mixed models (minimum of three timepoints of data) with random slopes and intercepts using the *lme4* package in R (56). For brain Aβ accumulation estimates, slopes were calculated using a least-squares linear regression fitted to a minimum of three timepoints of data, as previously described (57). The estimated slopes were assessed as the outcome measures using linear regression analyses in PLINK (53). Covariates for longitudinal analyses were the same as those in the cross-sectional analyses, with the addition of the intercept trait value (representing the individuals’ estimated value at baseline) also included as a covariate.

Following the linear regression analyses conducted separately for the two cohorts, the results were meta-analysed using the METAL software (54). The default METAL approach was applied, which combines p-values and effect directions from individual studies, weighted by sample size. To account for multiple testing, False Discovery Rate (FDR) correction was performed in R (58).

Glymphatic pathway genetic risk scores (G-GRS) were constructed using PRSice (55). Linear regression results from ADNI were transformed in a manner that corresponded to an increased β-coefficient associated with the effect (minor) allele representing an increased risk for each phenotype assessed. These results from ADNI were used as the discovery dataset, with the AIBL cohort used as the test dataset. The calculation of a participant’s GRS was done by summing each effect allele dosage weighted by the β-coefficient calculated in the linear regression analyses performed in the discovery cohort (ADNI) data. The resulting G-GRS values were then standardised within the target sample to have a mean of 0 and a standard deviation of 1. G-GRS were constructed for each outcome variable assessed (brain Aβ-burden, PACC and regional-brain volumes) under both cross-sectional and longitudinal models. This can be defined as:

$$\begin{array}{c} G-GRS_{i}=\sum EA_{n}\left(\beta_{n}\right)\\ G-GRS_{STD}=\frac{\sum EA_{n}\;\left(\beta_{n}\right)\;-\;Mean\;\left(G\;-\;GRS_{i}\right)}{SD\;\left(G\;-\;GRS_{i}\right)} \end{array}$$

Where n is each SNP included in the G-GRS, EA represents the effect allele under the specified genetic model (coded as 0, 1, or 2 based on the number of effect alleles under the additive model, and as presence or absence under the dominant and recessive models) and β is the converted estimated coefficient of the effect allele as determined by linear regression analyses in the ADNI cohort data. When constructing the G-GRS under the three genetic models (additive, dominant, and recessive), the results (β) from the corresponding linear regression analyses were used as the input. PRSice (55) was used to calculate the optimal p-threshold for the inclusion of SNPs into each G-GRS. In addition, PRSice was used to run linear regression analyses of these calculated G-GRS_STD_ against the appropriate outcome in the target AIBL dataset, with the inclusion of the same covariates as were in the original linear regression analyses with individual SNPs. Results were corrected for multiple comparisons using the FDR method (58). To visualise the relationship between the calculated G-GRS and outcome of interest the “*sjPlot*” package (59) in R was used.

## Results

### Demographics

The demographic characteristics of the ADNI and AIBL CU samples used in this study are summarised on Table 1. Following stratification of the CU cohorts by Aβ status primary analyses were conducted in the Aβ^+^ groups given their biological relevance. Findings for the Aβ^+^ groups are provided in the Supplementary Materials (Supplementary Tables 6–10). Participant sample sizes varied between the outcomes assessed, depending on data availability. For participants that had baseline Aβ data available and were classified as Aβ+ (*ADNI n = 404; AIBL n = 569*), ADNI participants were on average 1 year older compared to AIBL participants [Table 1]. For longitudinal analyses, participant inclusion was further restricted to those with a minimum of three timepoints of data available and therefore sample sizes once more differed depending on the outcome being assessed (*Aβ + with longitudinal Aβ data available ADNI n = 402; AIBL n = 562*). Sub-group sample sizes and demographic tables for individuals with the remaining outcome data available can be found in Supplementary Tables 1A-F, where ADNI participants were consistently older than AIBL participants (range 1–4 years), and had received more years of formal education on average.

### Genetic variants within astrocytic end-foot genes are associated with differences in AD-related phenotypes

**Figure 1.**
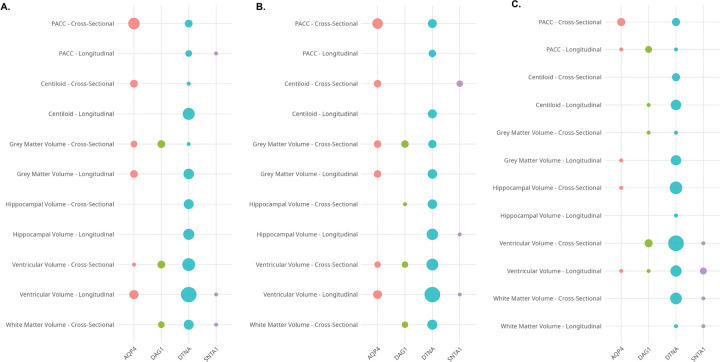
Bubble plots of meta-analysis of linear regression results For each of the outcomes assessed the number of SNPs that showed nominally significant associations within the four respective genes are represented by bubble size for (A) additive, (B) dominant and (C) recessive genetic models. Meta-analysis of linear regression results of SNPs run under additive genetic models. Abbreviations: SNP, single nucleotide polymorphism; PACC, Preclinical Alzheimer’s Cognitive Composite.

Meta-analyses of the linear regression results obtained from the AIBL and ADNI studies revealed significant (q < 0.05) associations between SNPs assessed under additive, dominant and recessive genetic models and AD-related phenotypes [Table 2; Fig. 1]. Of the ten significant associations identified, nine were cross-sectional. Under the additive genetic model, the association between one variant rs10502478, located upstream of *AQP4* and within its negative regulator *AQP4-AS1*, and cross-sectional meaures of PACC remained significant following FDR correction. The rs10502478 minor allele was associated with higher PACC scores *(Z Score = 3.702, p = 0.0002, q = 0.0293)*, and additionally was nominally associated with lower Aβ burden *(Z Score = −3.097,p = 0.0020, q = 0.2682)*. Under the dominant genetic model, four associations reached FDR significance [Table 2; Fig. 1]. The rs10502478 variant described above also remained significantly associated with cross-sectional PACC scores following FDR correction under the dominant genetic model *(Z Score = 3.913, p = 0.0001, q = 0.0125)*. In addition, the rs10164026 (*AQP4*) minor allele was associated with higher PACC scores *(Z Score = 3.38, p = 0.0007, q = 0.0497)*.

For cross-sectional ventricular volumes, carriage of the rs8092794 (*DTNA*) minor allele was associated with smaller volumes *(Z Score =−3.836 p = 0.0001, q = 0.0172)* under the dominant genetic model. Under the recessive genetic model [Table 2; Fig. 1], five variants remained significantly associated with cross-sectional ventricular volumes following FDR correction. Homozygosity of the rs13079082 (*DAG1*) minor allele was associated with smaller ventricular volumes *(Z Score = −3.46, p = 0.0005, q = 0.0206)*. Located within *DTNA*, homozygosity of rs17642885 *(Z Score = −3.37, p = 0.0008, q = 0.0206)* and rs79500711 *(Z Score = −3.812, p = 0.0001, q = 0.0189)* were both associated with smaller ventricular volumes. Meanwhile homozygosity of another two variants rs78837792 *(Z Score = 3.507, p = 0.0005, q = 0.0206)* and rs7233779 *(Z Score = 3.418, p = 0.0006, q = 0.0206)* within *DTNA1* were associated with larger cross-sectional ventricular volumes.

Finally, one association was seen in the longitudinal analysis. Here, under the dominant genetic model, carriage of the rs45556134 (*DTNA*) minor allele was associated with reduced rates of grey matter atrophy *(Z Score = 3.796, p = 0.0001, q = 0.0202)* and nominally associated with reduced hippocampal atrophy *(Z Score = 2.044, p = 0.0409, q = 0.5096)*. Full results tables are available in Supplementary Materials Tables S2–4.

### Glymphatic pathway genetic risk scores are associated with differences in AD-related phenotypes

**Figure 2.**
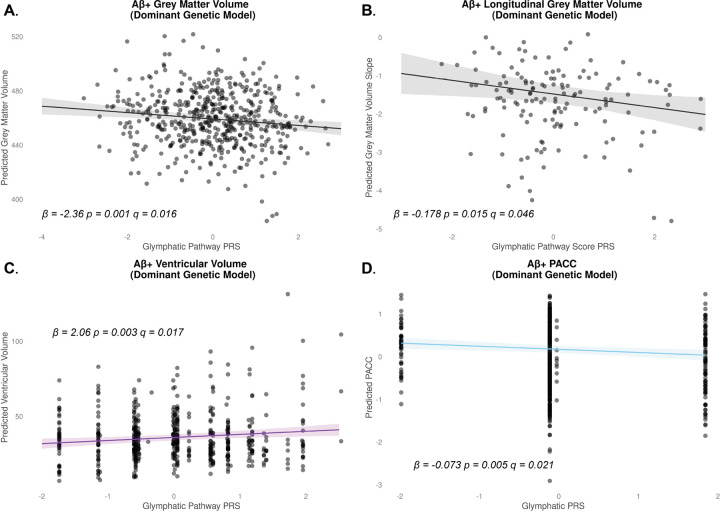
Glymphatic Pathway GRS (G-GRS) associations with AD-related phenotypes Representation of linear regression analyses visualising the relationships between the constructed Glymphatic Pathway GRS (G-GRS) that were associated with **(A)** cross-sectional grey matter volumes **(B)** longitudinal changes in grey matter volume **(C)**cross-sectional ventricular volumes and **(D)** cross-sectional PACC scores following FDR correction. Here, increasing scores for the G-GRS correspond to increasing genetic load of carriage of the ascertained risk alleles. G-GRS were derived from linear regression analyses undertaken using ADNI discovery dataset and the AIBL testing dataset in PRSice. The co-efficient is denoted as β, the p value represents the association between the G-GRS and phenotype and the q represents the False Discovery Rate corrected p value. Covariates: age, sex, *APOE-e4*carriage, education (PACC only) and intercept trait value (for longitudinal assessments only). Abbreviations: AIBL, Australian Imaging Biomarker and Lifestyle Study of Ageing; ADNI, Alzheimer’s Disease Neuroimaging Initiative; *APOE, apolipoprotein E;* GRS, genetic risk score; PACC, Preclinical Alzheimer’s Cognitive Composite.

Glymphatic pathway genetic risk scores (G-GRS) were constructed for each of the AD-related phenotypes assessed both cross-sectionally and longitudinally. These scores were constructed from the linear regression results of SNPs run under additive, dominant and recessive genetic models. Of the scores tested, ten (constructed under additive and dominant genetic models) were nominally associated with outcome measures in the test cohort AIBL [Table 3, Fig. 2]. Four of these G-GRS (all constructed and tested under a dominant genetic model) remained significant following FDR correction (*Cross-Sectional Grey Matter Volume: β= −2.359, SE = 0.72, p = 0.0011, q = 0.0136; Longitudinal Grey Matter Volume: β= −0.179, SE = 0.07, p = 0.0154, q = 0.0461; Cross-Sectional Ventricular Volume: β = 2.062, SE = 0.69, p = 0.0029, q = 0.0173; Cross-Sectional PACC: β= −0.073, SE = 0.03, p = 0.0052, q = 0.0208*) [Figure 2]. Full results tables are provided in Supplementary Materials Table S5.

## Discussion

This study examined associations between glymphatic pathway-related genes (*AQP4, DAG1, DTNA* and *SNTA1*) and multiple AD-related phenotypes using meta-analysed linear regression models and glymphatic pathway genetic risk scores (G-GRS). Robust associations between individual variants and cognition, grey matter and ventricular volumes were identified, and G-GRS for cross-sectional cognition, grey matter and ventricular volumes, and longitudinal grey matter volume remained significant after false discovery rate (FDR) correction.

At the single-variant level, one *DTNA* variant (rs45556134) was associated with grey matter atrophy, with carriage of the minor allele related to reduced atrophy rates, and six variants were associated with cross-sectional ventricular volumes, five (rs8092794, rs17642885, rs7233779, rs78837792 and rs79500711) in *DTNA* and one (rs13079082) in *DAG1*. To our knowledge, these variants have not been previously linked to brain health measures. The *DTNA* gene encodes for α-dystrobrevin protein, a member of the dystrophin family of proteins. In post-mortem regional brain analyses *DTNA* mRNA levels are increased in the hippocampal and temporal cortex regions of AD patients, as compared to healthy controls (25). In post-mortem human grey matter, there is a significant increase in α-dystrobrevin protein observed in the vessels of people with cerebral amyloid angiopathy, as compared to older healthy controls (21.71% vs 2.82%) (60). This upregulation may be compensatory, as α-dystrobrevin deficiency in animal models disrupts blood-brain barrier integrity and leads to brain oedema (61). The *DAG1* gene encodes dystroglycan 1, a transmembrane protein. In human post-mortem brains, increased *DAG1* mRNA *(t = 2.632, p = 0.013)* is seen in the hippocampus of people with dementia, and this increase is associated with increasing levels of phosphorylated tau protein, regardless of dementia status (25). In addition, higher levels of the α-dystroglycan protein have been reported in the cerebral spinal fluid of people with AD and Parkinson’s disease as compared to healthy controls (62). While this study is the first to associate *DAG1* and *DTNA* variants with brain phenotypes, the functional consequences of these specific variants for protein expression and localisation remain to be determined.

When cognition was assessed using PACC scores, two variants (rs10502478 and rs10164026) within or bordering *AQP4* were associated with cross-sectional differences, with minor allele carriage linked to better performance, and rs10502478 was also associated with lower Aβ levels. rs10502478 lies within an intron of *AQP4-AS1*, a long non-coding RNA upstream of *AQP4* that negatively regulates *AQP4* expression through antisense transcription, providing a plausible regulatory mechanism. To date, these specific variants have not been reported in relation to cognition or other AD phenotypes. Of the four genes assessed, only genetic variants within *AQP4* have been previously investigated in relation to cognition and Aβ (27, 29, 63, 64). Age-related mislocalisation of AQP4 from the perivascular endfeet, accompanied by increased expression, is exacerbated in AD and correlates with elevated cerebral Aβ and tau burden (15). Experimental ablation of *AQP4* in mouse models results in increased Aβ plaque deposition, synaptic dysfunction, cognitive impairment, neuroinflammation, and oxidative stress (as reviewed in (16)), while genetic variants in *AQP4* have been associated with cognitive decline, Aβ burden, and clinical progression of AD in humans (27, 29, 63, 64). The findings in the current study support associations between individual genetic variation in *AQP4* and differences in cross-sectional cognition.

Beyond single-variant effects, the study also assessed the cumulative impact of glymphatic pathway variation on AD-related phenotypes through the construction of a G-GRS. G-GRS showed robust associations with grey matter volumes assessed both cross-sectionally and longitudinally, as well as with cross-sectional ventricular volumes and cognitive measures. Previous studies have reported an average grey matter atrophy rate of between − 1.8 and − 2.62 cm^3^/year in healthy older adults (65–69), whilst in those with preclinical AD, a rate of − 6.99 cm^3^/year has been reported (70). In the current study, as the G-GRS increased, cross-sectional volumes decreased and the rate of grey matter atrophy also increased. The model estimated that each standard deviation increase in G-GRS predicts a 2.36 cm^3^ smaller volume cross-sectionally and an additional 0.178 cm^3^/year decline in grey matter volume, after adjusting for age, sex and *APOE-ε4* status, representing a modest effect. A relationship between glymphatic function and grey matter volume has been previously reported. Specifically, in a cohort of healthy older individuals, higher diffusion tensor imaging analysis along the perivascular space (DTI-ALPS) index measures (interpreted as higher glymphatic function) were associated with larger grey matter volumes, indicating a link between these regions and glymphatic functioning (71). A recent study also reported that the relationship between decreased ALPS index and worse cross-sectional cognition in a group of young onset AD patients was mediated by grey matter reserve, further suggesting a relationship between glymphatic function and grey matter (72).

Reduced glymphatic function as assessed through DTI-ALPS has also been associated with worse cognition in people with cerebral small vascular disease (73) and healthy controls (74). In this study the G-GRS for cognition showed a significant association with cross-sectional PACC scores. Here, as the G-GRS increased, PACC scores decreased. Specifically, each standard deviation increase in G-GRS predicted on average a 0.073 standard deviation lower PACC score.

Additionally, a significant association between the G-GRS and ventricular volumes was observed under a dominant genetic model. Biologically this represents that each standard deviation increase in G-GRS predicted an additional 2.06 cm^3^ in ventricular volume (dominant cross-sectional model). To put this in context, studies report that cognitively unimpaired older adults have average ventricular volumes of 37–38.3 cm^3^, and 45–55.3 cm^3^ for those with mild cognitive impairment (75, 76). In addition, a gain of 1.2–1.87 cm per year of ageing has been reported for individuals who are cognitively unimpaired (76, 77). Whilst our result is cross-sectional, this magnitude is slightly greater than what would typically be expected after one year of normal ageing.

The findings presented in this study are taken from cognitively unimpaired individuals who are classified as Aβ-positive, either having reached the threshold of positivity or on a positive trajectory of Aβ accumulation. Of note, most of the findings of significant relationships between G-GRS and regional brain volumes and cognition are in the absence of associations with Aβ burden, suggesting there may be other biological mechanisms that are leading to these downstream changes in brain phenotypes. For example, the glymphatic system can also mediate neuroinflammation through the carriage of immune cells and inflammatory mediators (78), which is known to contribute to brain atrophy and cognition. Aβ pathology may mediate these relationships, or alternatively may render the brain more susceptible to the downstream effects of glymphatic genetic variation. This interpretation is biologically plausible given the link between Aβ accumulation and glymphatic clearance, and is further supported by most of these reported associations not being observed in the Aβ-negative cohort (data in Supplementary Materials).

### Limitations

A limitation of the current study is that neither cohort has experimental measures of glymphatic clearance function. Therefore, whilst it is hypothesised that the genetic variation within these integral glymphatic genes may exert effects on the brain phenotypes through alterations to the glymphatic system, the functional consequences need to be examined in experimental settings. Examples of this include MRI-based assessments such as the calculation of the fractional volume of free water in brain parenchyma and diffusivity along the perivascular space index (79). Additionally, analyses were conducted in cohorts of predominantly European ancestry, which may limit the generalisability of findings to more diverse populations. Furthermore, as ADNI served as the discovery dataset for G-GRS construction and AIBL as the test dataset, the possibility of overfitting to ADNI-specific genetic effects in the G-GRS analyses cannot be entirely excluded; independent replication of the polygenic risk score findings in additional cohorts is therefore warranted.

## Conclusions

In conclusion, this study provides novel insights into the role of genetic variation within key glymphatic pathway-related genes in relation to AD-related phenotypes. By focusing on two cohorts of individuals who were clinically classified as Aβ + cognitively unimpaired at baseline, the study was able to detect associations relevant to the earliest changes in these markers. These findings align with prior literature linking glymphatic dysfunction to structural brain changes and cognitive decline, further supporting the hypothesis that impaired glymphatic clearance may contribute to neurodegenerative processes. Although experimental validation is needed to confirm the functional impact of these genetic variants on glymphatic activity, the present findings underscore the importance of the glymphatic system, and variation within its genetic architecture, as a potential contributor to neurodegeneration. Future studies integrating genetic, imaging-based, and functional assessments of glymphatic clearance will be essential to advancing our understanding of this pathway. Establishing the functional consequences of the variants identified here represents a critical next step in determining their relevance to AD pathogenesis.

## Supplementary Material

Supplementary Files

This is a list of supplementary files associated with this preprint. Click to download.

• SuppMaterialsTables15AB.xlsx

• SuppMaterialsTables610AB.xlsx

• SupplementaryFigure1.jpg

## Figures and Tables

**Table 1:** Aβ + Participant Demographics

n	ADNI	AIBL	p
	404	569	
**Age mean(sd)**	74 (6.7)	72.9 (6.6)	**0.01**
**Sex**			0.57
**Male**	177(43.8%)	261(45.9%)	
**Female**	227(56.2%)	308(54.1%)	
**APOE-e4**			0.97
**ε4-**	247(61.1%)	346(60.8%)	
**ε4+**	157(38.9%)	223(39.2%)	
**Centiloid mean(sd)**	29.4 (35.8)	33.5 (38.9)	0.09

Baseline participant demographics of individuals with baseline Aβ measures. Sub-group participants demographic tables can be found in Supplementary Tables 1A-F. Abbreviations: AIBL, Australian Imaging Biomarker and Lifestyle Study of Ageing; ADNI, Alzheimer's Disease Neuroimaging Initiative; APOE, apolipoprotein E; PACC, Preclinical Alzheimer's Cognitive Composite.

**Table 2:** Significant Associations from Meta Analysis

Outcome	Model	Model	Gene Symbol	SNP	CHROM	POS	A1	A2	Weight	Zscore	Direction	P	FDR P
PACC	Cross-Sectional	ADD	*AQP4*	rs10502478	18	26874616	A	C	964	3.702	++	**0.000214**	**0.0293**
PACC	Cross-Sectional	DOM	*AQP4*	rs10164026	18	26842362	A	C	964	3.38	++	**0.000725**	**0.0497**
PACC	Cross-Sectional	DOM	*AQP4*	rs10502478	18	26874616	A	C	964	3.913	++	**0.000091**	**0.0125**
Grey Matter Volume	Longitudinal	DOM	*DTNA*	rs45556134	18	34729425	A	G	309	3.796	++	**0.000147**	**0.0202**
Ventricular Volume	Cross-Sectional	DOM	*DTNA*	rs8092794	18	34496273	A	G	846	−3.836	−−	**0.000125**	**0.0172**
Ventricular Volume	Cross-Sectional	REC	*DAG1*	rs13079082	3	49537375	C	G	846	−3.46	−−	**0.000539**	**0.0206**
Ventricular Volume	Cross-Sectional	REC	*DTNA*	rs17642885	18	34649976	T	C	846	−3.37	+−	**0.000750**	**0.0206**
Ventricular Volume	Cross-Sectional	REC	*DTNA*	rs7233779	18	34863262	A	G	846	3.418	++	**0.000632**	**0.0206**
Ventricular Volume	Cross-Sectional	REC	*DTNA*	rs78837792	18	34703359	T	C	846	3.507	++	**0.000453**	**0.0206**
Ventricular Volume	Cross-Sectional	REC	*DTNA*	rs79500711	18	34794379	A	G	518	−3.812	?−	**0.000138**	**0.0189**

Meta-analysis of linear regression results of SNPs significantly associated (FDR p < 0.05) with AD-related phenotypes. Covariates: age, sex, *APOE-e4* carriage and intercept trait value (for longitudinal assessments only). Abbreviations: ADD, additive genetic model; *APOE, apolipoprotein E;* CHROM, chromosome; A1, effect allele; A2, reference allele; DOM, dominant genetic model; FDR, False Discovery Rate; PACC, Preclinical Alzheimer's Cognitive Composite; REC, recessive genetic model; SNP, single nucleotide polymorphism.

**Table 3:** Glymphatic Pathway GRS (G-GRS) Results

Oucome		Genetic Model	SNP	P Threshold	GRS	Full	Null	β	SE	P	FDR P
			(N)		R2	R2	R2				
PACC	CS	DOM	2	0.005	0.010	0.287	0.276	−0.073	0.03	**0.0052**	**0.0208**
Grey Matter Volume	CS	ADD	23	0.220	0.011	0.236	0.225	−1.979	0.72	**0.0060**	0.0606
Grey Matter Volume	CS	DOM	30	0.360	0.016	0.240	0.225	−2.359	0.72	**0.0011**	**0.0136**
Grey Matter Volume	Long	ADD	116	1.000	0.035	0.152	0.118	−0.172	0.07	**0.0202**	0.0606
Grey Matter Volume	Long	DOM	49	0.432	0.038	0.155	0.118	−0.179	0.07	**0.0154**	**0.0461**
Ventricular Volume	CS	ADD	5	0.096	0.010	0.131	0.122	1.696	0.71	**0.0166**	0.0606
Ventricular Volume	CS	DOM	7	0.044	0.015	0.137	0.122	2.062	0.69	**0.0029**	**0.0173**
Ventricular Volume	Long	ADD	58	0.412	0.026	0.375	0.349	0.129	0.05	**0.0185**	0.0606
Ventricular Volume	Long	DOM	61	0.390	0.023	0.372	0.349	0.120	0.05	**0.0276**	0.0663
White Matter Volume	CS	ADD	38	0.281	0.008	0.087	0.079	2.211	1.02	**0.0312**	0.0748

Glymphatic pathway GRS (G-GRS) that were nominally significant (p < 0.05) that were constructed and tested under additive, dominant and recessive genetic models. For full see Supplementary Table 5. Abbreviations: ADD, additive genetic model; CS, cross-sectional; DOM, dominant genetic model; FDR, False Discovery Rate; GRS, genetic risk score; Long, longitudinal; PACC, Preclinical Alzheimer's disease Cognitive Composite; REC, recessive genetic model; SE, standard error; SNP (n); number of single nucleotide polymorphisms included in the GRS.

## Data Availability

AIBL data analysed during the current study are publicly available through an expression of interest procedure (https://aibl.org.au/collaboration/). ADNI data, and a subset of AIBL data, are also publicly available through an application procedure (https://adni.loni.usc.edu/).

## List of Abbreviations

AD: Alzheimer’s disease
ADNI: Alzheimer’s disease Neuroimaging Initiative
AIBL: Australian Imaging Biomarker and Lifestyle Study of Ageing
APOE: Apolipoprotein E
AQP4: aquaporin 4
Aβ: amyloid-β
BBB: blood brain barrier
CSF: cerebrospinal fluid
DAC: dystrophin-associated complex
DAG1: dystroglycan
DMD: dystrophin
DTI-ALPS: diffusion tensor imaging analysis along the perivascular space
DTNA: dystrobrevin
EA: effect allele
FDR: False Discovery Rate
G-GRS: glymphatic pathway genetic risk score
MPRAGE: magnetisation-prepared rapid gradient echo
MRI: magnetic resonance imaging
PACC: Preclinical Alzheimer’s Cognitive Composite
PET: positron emission tomography
QC: quality control
SNP: single nucleotide polymorphism
SNTA1: α-syntrophin
SUVR: standardised uptake value ratios
TOPMed: Trans-Omics for Precision Medicine
